# Historical review of the List of Occupational Diseases recommended by the International Labour organization (ILO)

**DOI:** 10.1186/2052-4374-25-14

**Published:** 2013-08-05

**Authors:** Eun-A Kim, Seong-Kyu Kang

**Affiliations:** 1 Occupational Safety and Health Research Institute, Korea Occupational Safety and Health Agency, Incheon (403-711), Korea; 2 Seoul Regional Office, Korea Occupational Safety and Health Agency, Seoul (156-754), Korea

**Keywords:** Occupational disease, List, Compensation

## Abstract

The list of occupational diseases established in the international and national legal system has played important roles in both prevention of and compensation for workers’ diseases. This report reviewed the historical development in the ILO list of occupational diseases and suggested implications of the trends. Since the first establishment of the ILO list of occupational diseases in 1925, the list has played a key role in harmonizing the development of policies on occupational diseases at the international level. The three occupational diseases (anthrax, lead poisoning, and mercury poisoning) in the first ILO list of occupational diseases, set up in 1925 as workmen’s compensation convention represented an increase of occupational diseases from the Industrial Revolution. Until the 1960s, 10 occupational diseases had been representative compensable occupational diseases listed in Convention No. 121, which implies that occupational diseases in this era were equated to industrial poisoning. Since 1980, with advancements in diagnostic techniques and medical science, noise-induced hearing loss, and several bronchopulmonary diseases have been incorporated into the ILO occupational list. Since 2002, changes in the structure of industries, emerging new chemicals, and advanced national worker’s compensation schemes have provoked the ILO to revise the occupational disease list. A new format of ILO list appended in Recommendation 194 (R194) was composed of two dimensions (causes and diseases) and subcategories. Among 50 member states that had provided their national lists of occupational diseases, until 2012 thirty countries were found to have the list occupational diseases having similar structure to ILO list in R194.

## Introduction

The list of occupational diseases is a collection of diseases caused by exposure during work. The list contains the definition of each occupational disease and is basis on the fundamental occupational safety and health legislation. The list of occupational diseases officially recognized by the international and national legal systems plays important roles both in prevention of and compensation for workers’ diseases. Since the establishment of occupational diseases as compensable diseases in the Workers’ Compensation Act in Germany [1], this list of occupational diseases has come to be regarded as a criterion for compensable diseases in the United States [2], United Kingdom, and many other European countries following the German insurance system [3].

Most workers’ compensation legislation includes the core phrases of the definition of occupational diseases; it then goes on to provide guidelines for identifying, reporting, and registering of occupational diseases. This required data collection forms the basis of prevention strategies. Thus, the officially enacted list of occupational diseases has an impact not only on the provision of employment injury benefits, but also on national and enterprise level preventive programs. From its year of establishment in 1919, the International Labour Organization (ILO) concerned itself with the management of occupational diseases, beginning with 2 diseases in its formative years. The official list of occupational disease was established later for the sake of workers compensation convention [4]. Since then, the ILO led its member states in terms of the scope of occupational disease by providing them with a recommended list of common occupational diseases convention or recommendation [5], which had been established by consensus through a tri-party (government, employer and employee) debate.

Since the predominance of occupational diseases has been changing due to transformations in the structure of industry and working conditions, the ILO List of Occupational Diseases has been revised. The revisions have been influenced by the modernization of industry and by other international organizations as well as the European Union, and it has also been affected by the development and revision of the national occupational lists. Each national occupational list, usually presented as a schedule in the workers’ compensation law, presents a large variety of concepts for covering occupational diseases within the workers’ compensation system, reflecting the social, cultural, and technological background and environment of the system [6]. In 2002, based on Recommendation No. 194 (R194), the ILO List of Occupational Diseases was modified to emphasize prevention and reporting more than compensation [7]. The implications of those changes can be traced through each era, by reviewing the changing definition of occupational diseases and the expansion of work-relatedness over time. In this report, we traced of the historical development of the ILO List of Occupational Diseases and what the changes in the list imply.

## Review

### *Era of industrial poisoning reflected in the ILO List*

Anthrax and lead poisoning were the first occupational diseases by the ILO (Table 1) as recommendation No 3 (R03) [8] and recommendation No.4 (R04) [9] when the organization was established in 1919. R03 called for the protection of workers in the mass production system from anthrax infection, while R04 demanded the protection of vulnerable workers from industrial poisoning.

**Table 1:** Occupational disease list of the ILO during the industrial poisoning era

**ILO legislation**	**R03, R04 (1919)**	**C18 (1925)**	**C42 (1934)**
Chemical	1) Lead	2) Mercury	3) Phosphorous, 4) arsenic, 5) Benzene or its homologues, their nitro- and amino-derivatives, 6) halogen derivatives of hydrocarbons of the aliphatic series
Physical			1) Radium and other radioactive substances, 2) X-rays
Biological	1) Anthrax	No change	No change
Pulmonary			1) Silicosis with or without pulmonary tuberculosis, provided that silicosis is an essential factor in causing the resultant incapacity or death.
Cancer			1) Primary epitheliomatous cancer of the skin.

*ILO*: International Labour Organization, *R03*: ILO recommendation No.3, *R04*: ILO recommendation No. 4, *C18*: ILO convention No. 18, *C42*: ILO convention No. 42.

Although anthrax was first reported as early as 1250 BC, the Industrial Revolution was responsible for placing anthrax at center stage [10]. Anthrax was frequently found in English woolen mill workers exposed to contaminated hides and wool, from which its other name, woolsorter’s disease, is derived. This problem brought to bear the competing philosophies of the day: the unregulated free market versus the safety- and health-regulated workplace [10].

The huge increase in demand for lead engendered by the Industrial Revolution brought about the problem of industrial diseases, of which the most widespread was lead poisoning [11]. Women and children were employed in all stages of lead processing, including the highly dangerous jobs of pottery glazing, smelting of lead ores, and manufacturing of lead compounds, particularly white lead [12]. After 1900, as a result of intensive industrial hygiene studies, many countries passed legislation regarding the protection of lead workers [11].

In 1925, Workmen’s Compensation (Occupational Diseases) Convention No. 18 (C18), which was established as the first ILO List of Occupational Diseases, comprising two industrial poisonings (lead and mercury) and one infection (anthrax) [13], emphasized the workers’ right to compensation. Then, years later, the convention was revised to Convention No. 42 (C42) [14]. The new list of occupational diseases added 7 additional diseases to the previous list of lead poisoning, mercury poisoning, and anthrax infection. The newly added diseases included 4 types of poisoning;phosphorus poisoning, arsenic poisoning, poisoning by benzene, and poisoning by halogen derivatives of hydrocarbons of the aliphatic series, and 3 diseases; silicosis, diseases due to radiation, and skin cancer (Table 1). The 10-item list was maintained for 30 years, until it was revised as Employment Injury Benefits Convention No. 121 (C121) in 1964 [15], adding 4 more poisoning items (manganese, chromium, beryllium, and carbon bisulfide) and rephrasing X-rays and radon to “ionizing radiation” (Table 2). Until then, the ILO List of Occupational Diseases was composed of obvious occupational diseases, such as the poisoning of a few obvious chemicals in large modern industries, the classic lung disorder of the mining industry (pneumoconiosis), and occupational skin cancer first identified in 1775 [16], because the ratifying countries shall, at minimum, recognize the occupational origin of all the diseases in this list [4]. Although the ILO List of Occupational Diseases up to C121 (1964) has the disadvantage of covering a limited number of occupational diseases, it has the advantage of listing only diseases for which there can be a presumption that they are of occupational origin. Specifically, paragraph 6-(2) of Recommendation No. 121 provides that “unless proof to the contrary is brought, there should be a presumption of the occupational origin of such diseases.” [7] Because of this presumption, the ILO List has a paragraph mentioning the condition by which a specific disease is recognized, such as “silicosis causing the resultant incapacity or death.” (Table 2)

**Table 2:** New items on the occupational disease list in Convention No. 121

**ILO legislation**		**C121 (1964, revised 1980)**
Chemical	‘64	6) Beryllium, 7) Chromium, 8) Manganese, 9) Carbon disulfide
	‘80	10) Toxic halogen derivatives of aliphatic or aromatic hydrocarbon, 11) Cadmium, 12) Arsenic, 13) Fluorine, 14) Nitroglycerin or other nitric acid esters, 15) Alcohols, glycols or ketones, 16) Asphyxiants: carbon monoxide, hydrogen cyanide, or its toxic derivatives, hydrogen sulfide
Physical	‘64	1) Ionizing radiation
	‘80	2) Hearing impairment caused by noise, 3) diseases caused by vibration, 4) diseases caused by work in compressed air
Biological	‘80	1) Infectious or parasitic diseases contracted in an occupation where there is a particular risk of contamination (health or laboratory work, veterinary work, animal handling work, other work with contamination risk)
Pulmonary	‘80	1) Pneumoconiosis caused by sclerogenic mineral dust (silicosis, anthracosilicosis, asbestosis) and silicotuberculosis, provided that silicosis is an essential factor in causing the resultant incapacity or death, 2) Bronchopulmonary diseases caused by hard-metal, 3) Bronchopulmonary diseases caused by cotton dust (byssinosis), or flax, hemp, or sisal dust, 4) Occupational asthma, 5) Extrinsic allergic alveolitis and its sequelae caused by the inhalation of organic dusts, as prescribed by national legislation
Skin	‘80	1) Skin diseases caused by physical, chemical, or biological agents
Cancer	‘80	2) Lung cancer or mesotheliomas caused by asbestos

*ILO*: International Labour Organization, *C121*: ILO convention No. 121.

#### Expansion of the ILO List of Occupational Diseases beyond poisoning

In 1980, C121 was revised again with growing public awareness about occupational diseases [17]. However, this time, the change was not about simply adding a few more items. The revision of C121 not only added 7 more types of chemical poisoning, respiratory disease, and disorders caused by physical agents, but also expansion its scope to skin disease and infectious disease (Table 2). The causal relationship of most cases of poisoning is relatively strong, such that non-occupational factors can be excluded. On the other hand, non-poisoning diseases, such as noise-induced hearing loss, most bronchopulmonary diseases, or infectious diseases, are common in the general population; hence, it is difficult to differentiate these occupational diseases from non-occupational ones, except for in a few cases. Further, most cases are related to chronic, long-term exposure.

Although hearing impairment caused by noise is stated to be the most prevalent industrial disorder in the most recent occupational medicine textbooks [18], it is only within the past 40 years that serious attention has been paid to the excessive noise at work sites. With the Industrial Revolution, the development of metal industries and the steam engine created a high level of noise. However, studies regarding the specific mechanism by which noise affects hearing, along with reliable methods of measuring hearing and the ambient noise level were not developed until the beginning of the twentieth century. Even as late as in 1950, a monograph on noise [19] noted that much of the published information about the effects of noise on human health was either “unsupported opinion” or was derived from “poorly designed experiments.” For example, during the early part of the twentieth century in the United States, before the compensation system was expanded, only hearing loss from an instantaneous injury, such as an explosive blast, was deemed to be compensable, not gradually developed hearing loss [20]. Therefore, the inclusion of noise-induced hearing loss on the ILO List of Occupational Diseases was highly challenged until 1980.

Another challenge to the ILO List of Occupational Diseases was related to several textile-related bronchopulmonary diseases caused by dust and organic dust, occupational asthma and extrinsic allergic alveolitis. These conditions have been reported since the early eighteenth century [21]; however, their recognition was not easy until, developments in immunology, spirometry, and pulmonary pathophysiology in the twentieth century played a key role in their evolution as clinical entities [22].

By the end of March 2013, 24 countries have ratified C121 [23]. A number of countries have their own equivalent Schedule 2, which is almost always based heavily on the ILO List [24]. ILO C121 (revised 1980) reflected the major occupational diseases beyond industrial poisoning and the scientific advances of the era.

#### Further updates to the ILO List appended to R194 through tripartite meetings

The List of Occupational Diseases in C121 amended in 1980 reflected the knowledge of the 1970s. Since then, there have been several significant changes in the structure of industry (transitioning from heavy industries to service fields), workplace risk (emerging new industrial chemicals), and compensation policy. A number of occupational cancers have been recognized and included in various national compensation schemes [25-32]. Furthermore, the 12 countries of the European Communities issued a comprehensive recommendation for the European Schedule of Occupational Diseases (EU List) on May 1990 [33], which would be revised again in 2003 [34] by developing more comprehensive contents than the ILO List appended to C121. The EU List in 1990 included 24 additional chemicals not in the ILO List. Nine chemicals were listed as causes of occupational skin cancer, and eight musculoskeletal disorders that had not yet appeared in the ILO List as of C121 were also included in the EU list (Table 2).

This situation provoked the ILO’s revision of its occupational disease list in 1990–1991 by reviewing the law and practice of occupational diseases in the varying national legislation of the ILO member states, and of the current practices in diagnosis, reporting, and evaluation for compensation purposes, as well as by taking account of the lists in force along with national practices in 76 different states or countries [4]. The expert group recommended a new format for the ILO List of Occupational Diseases, which is now composed of three categories: diseases by agents (chemical, physical, biological), diseases of a target organ (respiratory, skin, and musculoskeletal), and occupational cancer [4,35]. Sixteen chemicals, 2 physical agents, 4 pulmonary disorders, and one skin disease were added to the list (Table 3). The occupational cancer category comprised 14 carcinogens, of which the inclusion criterion was the category 1 agents listed by the International Agency for Research on Cancer (IARC) [4]. The categories of disease by agents, pulmonary disease, and occupational cancer contain a flexible clause, which is stated as “other diseases, not mentioned in the list.” Musculoskeletal disease is also listed with the general definition of work-relatedness (Table 3).

**Table 3:** New items on the occupational disease list of Recommendation No. 194

**ILO legislation**		**R194 (2002, revised 2010)**
Chemical	‘02	17) Acrylonitrile, 18) Oxide of nitrogen, 19) Vanadium , 20) Antimony, 21) Hexane, 22) Mineral acid, 23) Thallium, 24) Pharmaceutical agents, 25) Osmium, 26) Selenium, 27) Copper, 28) Platinum, 29) Tin, 30) Zinc, 31) Ozone, phosgene, 32) Corneal irritants like benzoquinone, 33) Diseases caused by other chemical agents at work not mentioned
	‘10	34) Nickel, 35) ammonia, 36) isocyanates, 37) pesticides, 38) sulfur oxides, 39) organic solvents, 40) latex, 41) chlorine
Physical	‘02	5) Disease caused by heat radiation, 6) Disease caused by ultraviolet radiation, 7) Disease caused by exposure to extreme temperatures, 8) Other physical agents not mentioned
	‘10	Modified 6) to “diseases caused by optical (ultraviolet, visible light, infrared) radiations including laser”
Biological	‘02	No change (2002)
	‘10	1) Brucellosis, 2) Hepatitis viruses, 3) HIV, 4) Tetanus, 5) Tuberculosis, 6) Toxic or inflammatory syndrome associated with bacterial or fungal contaminants, 7) Anthrax, 8) Leptospirosis, 9) Infectious disease not mentioned
Pulmonary	‘02	6) Siderosis, 7) Chronic obstructive pulmonary diseases caused by inhalation of coal dust, dust from stone quarries, wood dust, dust from cereals and agricultural work, dust in animal stables, dust from textiles, and paper dust, 8) Diseases of the lung caused by aluminum, 9) Upper airway disorders caused by recognized sensitizing agents or irritants, 10) Other respiratory diseases not mentioned
	‘10	10) Pneumoconiosis caused by non-fibrogenic mineral dust
Skin	‘02	2) Vitiligo caused by other recognized agents
	‘10	3) Allergic contact dermatoses and contact urticaria, 4)Irritant contact dermatoses caused by other recognized irritant agents, 5)Other skin diseases caused by physical, chemical, or biological agents
Cancer	‘02	1) Asbestos, 2) Benzidine and its salts, 3) BCME, 4) Chromium VI, 5) Coal tars, coal tar pitches, 6) Beta-naphtylamine, 7) vinyl chloride, 8) Benzene, 9) Toxic nitro and amino derivatives of benzene or its homologue, 10) Ionizing radiation, 11) Tar, pitch, bitumen, mineral oil, anthracene, or compounds, 12) Coke oven emissions, 13) Nickel, 14) Wood dust, 15) Other carcinogens
	‘10	16) Arsenic, 17) Beryllium, 18) Cadmium, 19) Erionite, 20) Ethylene oxide, 21) Hepatitis B virus and C virus
MSD	‘02	1) Musculoskeletal diseases caused by specific work activities or work environments where particular risk factors are present (rapid or repetitive motion, forceful exertion, excessive mechanical force concentration, awkward or non-neutral postures, vibration, local or environmental cold may potentiate risk)
	‘10	1) Radial styloid tenosynovitis of the wrist, 2) Chronic tenosynovitis of the wrist, 3) Olecranon bursitis, 4) Prepatellar bursitis, 5) Epicondylitis, 6) Meniscus lesions, 7) Carpal tunnel syndrome, 8) Other musculoskeletal disorders not mentioned
Mental and behavioral	‘10	1) Post-traumatic syndrome, 2) Other mental and behavioral disease

*ILO*: International Labour Organization, *R194*: ILO recommendation NO. 194, *MSD*: musculoskeletal disorder.

However, the procedure for amending the List of Occupational Diseases requires a difficult process, placement on the agenda of the International Labour Conference, and adoption by a two-third’s majority. Due to competing priorities, the revision of the ILO List could not be placed on the agenda until the 90^th^ Session of the International Labour Conference in 2002, when R194, the new ILO recommendation concerning the List of Occupational Diseases and the Recording and Notification of Occupational Accidents and Diseases, was adopted [36], leaving C121 (1980) unchanged. The purpose of R194 emphasizes its role as a tool for reporting, notifying, and identifying the causes of occupational diseases rather than supporting the compensation system [36]. Moving the occupational disease list from the Compensation Convention (C121) to the Recommendation for Recording and Notification (R194) gave more flexibility in developing a more comprehensive list. Although the Convention and Recommendation are both international labor standards, the adoption of a convention requires ratification by member countries, by which a convention comes into force for that country one year after the date of ratification.

The new ILO List appended to R194 was revised again through two tripartite meetings of experts in 2005 and 2009. Preparing this meeting, the ILO analyzed 50 of the most up-to-date national and other lists of occupational diseases, including the EU Schedule of Occupational Diseases [37]. Further, it collected 160 replies to a questionnaire from 80 governments of the member states [38] and the World Health Organization (WHO) and the International Commission on Occupational Health (ICOH) regarding the occupational disease list [7,37].

During tripartite meetings, the ILO prepared a technical background report regarding new items to be included in the ILO R194 list in 2005, [39] which the representatives of the government, employers, and workers discussed. The new items proposed by the ILO were 5 chemicals, 1 physical agent, 5 biological agents, 2 skin diseases, 7 musculoskeletal disorders, 2 mental and behavioral disorders, and 8 occupational carcinogens [39]. Although the majority of the replies to the questionnaire were affirmative of the proposed list with some additional comments [38], some items, such as disease due to radiofrequency radiation, cancer caused by formaldehyde, cancer caused by silica, and psychosomatic psychiatric syndromes caused by mobbing, were not accepted to the final list due to other representatives’ objections (Table 4) [38,40]. Representatives of the governments and workers also proposed several new items for the list during the tripartite meeting; however, an agreement could not reached on most of them [41] (Table 4). The most frequent objection from the representatives of employers was deletion of the items regarding “any other items not mentioned in the proceeding part” in every section. Finally, the ILO Governing Body at its 307^th^ Session, which took place in March 2010, approved the updated new list, which replaces the preceding one approved in 2002, comprising 41 chemicals, 7 physical agents, 9 biological agents, 12 respiratory diseases, 4 skin diseases, 8 musculoskeletal diseases, and 2 mental and behavioral disorders [42].

**Table 4:** Unaccepted proposals during the tripartite meeting in 2005 and 2009

		**R194 (2002, revised 2010)**
Chemical	Government	Add acids, aldehydes, organochlorinated or organophosphorous pesticides
	Employer	1. Delete oxides of nitrogen, pharmaceutical agents, osmium, selenium, copper, tin, zinc, and ozone
		2. Delete “ diseases caused by any other chemical agents not mentioned”
	Worker	Add “disease to endocrine system due to chemical agents”
Physical	ILO	Add “disease due to radiofrequency radiation”
	Employer	1. Delete ultra-violet radiation
		2. Delete “Diseases caused by any other physical agents not mentioned”
	Worker	Add “disease caused by electromagnetic radiation”
Biological	Government	Add “disease caused by enzymes”
	Worker	Add “intoxications where a direct link between the exposure of a worker to biological agents and the diseases is established”
Pulmonary	Government	Delete “chronic obstructive pulmonary diseases”
	Employer	1. Delete “chronic obstructive pulmonary diseases”
		2. Delete “any other respiratory disease not mentioned”
	Worker	1. Add “any other respiratory diseases caused by asbestos not covered in the preceding item”
		2. Add “bronchopulmonary diseases caused by synthetic or man-made fibers”
Skin	Government	Add “dermatoses of allergic origin”, and mycosis
Cancer	ILO	Add “cancer caused by formaldehyde” and “cancer caused by silica”
	Government	Delete “dust from wood”
	Employer	1. Insert specific cancer name for every carcinogen
		2. Delete “cancer caused by any other agents not mentioned”
	Worker	Add “Silica, crystalline in the form of quartz or cristobalite”
MSD	Worker	1. Add “any other musculoskeletal disorders not mentioned in 2.3.1 due to occupational psychological factors including mental fatigue”
		2. Add “central nervous system disorders of occupational origin”
Mental and behavioral	ILO	Add “psychosomatic psychiatric syndromes caused by mobbing”
	Worker	1. Add “disabling occupational neuroses”, 2. add “professional laryngitis with aphonia”, 3. add “diseases of a physical or psychological nature related to violence arising out of or in the course of employment”

*ILO*: International Labour Organization, *MSD*: musculoskeletal disorder.

#### The structures of the occupational disease lists in ILO member states

Among 50 member states that had provided their national lists of occupational diseases to prepare new ILO list in 2005, until 2012, 30 of these countries were found to have an occupational disease list with a similar structure to the ILO list in R194, composed of a causes part and diseases part [43]. Chemically induced disease was the common category, appearing in the lists of all of the 30 countries, including 8–65 chemicals. The second most common category was biological agents (22 countries), followed by physical agents (22 countries), respiratory diseases (20 countries), skin disorders (19 countries), and cancers (13 countries) (Table 5).

**Table 5:** Structure of the lists of occupational diseases in ILO member states

	**Categories(number of countries)**	**Asia-Pacific(number of items)**	**Africa (number of items)**	**Europe (number of items)**	**Americas (number of items)**
Causes	Chemicals (30)	China(56), Hong Kong(22), Japan(*), Malaysia(10), Saudi Arabia(40), Taiwan(45), Vietnam(8)	Algeria(56), Angola(31)	Belgium(15), Bulgaria(57), Czech(55), Denmark(29), Finland(36), German(31), Hungary(56), Ireland(30), Luxemburg(27), Russia(38), Portugal(31), Spain(45), Swiss(61), Turkey(65), UK(32)	Chile(18), Costa Rica(36), El Salvador(32), Mexico(36), Nicaragua(45)
	Physical agents (22)	China(5), Hong Kong(9), Japan(13), Saudi Arabia(7), Vietnam(4)	Algeria(12), Angola(11)	Belgium(17), Bulgaria(12), Czech(12), Finland(6), German(15), Hungary(8), Ireland(15), Portugal(11), Rumania(11), Russia(12), Spain(12), Turkey(14), UK(14)	Chile(5), Nicaragua(12), El Salvador(9), Mexico(11)
	Biomechanical	Japan(5)		Bulgaria(3), Finland(3),	
	Biological (28)				
	Job categories (8)	Japan(5)		Czech(3), Denmark(3), Finland(3), German(4), Russia(3)	Spain(4), Nicaragua(4)
	Pathogens(19)	China (3), Vietnam(3), Malaysia(7), Hong Kong(12), Saudi Arabia(17), Taiwan(9), Vietnam(3)	Angola(42)	Rumania(6), Belgium(28), Bulgaria(32), Hungary(18), Ireland(10), Portugal(4), Turkey(30), UK(15)	Chile(10), El Salvador(13), Mexico(21)
	Possible agents (3)			Rumania(19) Spain(40)	Nicaragua(40)
					
Diseases	Respiratory (20)	Malaysia(9), Saudi Arabia(20) Taiwan(6), Vietnam(4)	Angola(5)	Belgium(29), Czech(11), German(16), Portugal(5), Rumania(25), UK(13), Denmark(13)	Colombia, Costa Rica(17), El Salvador(17), Mexico(17)
	Pneumoconiosis (6)	China (13), Japan(5)		Ireland(1), Mexico(30)	Costa Rica(3), El Salvador(22)
	Skin (19)	China(8), Malaysia(11), Saudi Arabia(6), Taiwan(2), Vietnam(2)	Angola(21)	Belgium(9), Czech(1), Denmark(2), German(2), Portugal(22), Rumania(7), Spain(4), Turkey(2)	Costa Rica(31), El Salvador(16), Nicaragua(4), Mexico(18)
	Allergic (3)		Angola(15)	Portugal(6), Russia(16)	
	Musculoskeletal (3)	Saudi Arabia(7)		Rumania(12) Denmark(20)	
	Industrial fatigue(2)				El Salvador(3), Mexico(6)
	Cancer (13)	China(8), Japan(18), Saudi Arabia(18), Taiwan(18)	Angola (10)	Portugal(10), Russia(7), Rumania(18+IARC), Spain(28) Denmark(23)	El Salvador(3), Mexico(4), Nicaragua(28)

*: specific number is not available.

Most of the diseases related to ergonomic causes were categorized as physical agents, except in 3 countries. Eight countries categorized disorders related to biological agents according to risky jobs or industries, while 19 other countries listed specific pathogens. Pneumoconiosis was found to be a separate category in 6 countries (Table 5).

Although the majority of ILO member states adopted the broad structure of the ILO List in R194, each country’s list contains some specific occupational disease items not included in ILO List of R194 [43] (Table 6). Phenol derivatives are representative chemical items not presented in the ILO List. Several carcinogenic agents, such as aflatoxin, anticancer drugs, and silica, and well-known occupational biological pathogens such as rickettsia, malaria, and ameba are also not included in the ILO List. The most prominent difference of ILO 194 from some countries mentioned in Table 6 involved items regarding cardio-cerebro-vascular disorders induced by psychological stress, neurosis, and low back pain (Table 6).

**Table 6:** Examples of occupational disease from each country that are not included in the ILO’s List of Occupational Diseases R194

**Categories**	**Items (countries)**
Chemical agents	Phenol derivatives (Austria, Belgium, China, Costa Rica, El Salvador, Finland, Romania, Swiss, Turkey, France)
Carcinogenic agents	Aflatoxins (Denmark, Finland), Aliphatic aromatic and alicyclic hydrocarbons (Finland), lead (Denmark), trichloroethylene (Denmark), benzidine dye (France), ortho-toluidine (France), anticancer drugs (Finland), Aluminum production process (Denmark), 4-nitrodiphenyls (Canada, Japan), trichloroethylene (Denmark), tetrachloroethylene (Denmark), 2,3,7,8,-TCDD (Denmark), silica (German, United Kingdom, Romania, Taiwan), formaldehyde (Denmark, Malaysia, Taiwan), lead (Denmark, Saudi Arabia), leather (Ireland, Italy, Saudi Arabia, United Kingdom), iron mining with radon exposure (Denmark)
Biological agents	Rickettsia (United Kingdom, Russia, Philippines, Mexico, Nicaragua, Portugal, Saudi Arabia, Spain, Austria), Streptococcus (Algeria, Angola, France, Mexico, Portugal, Romania, Saudi Arabia, United Kingdom, New Zealand, Hong Kong), Thermophilic actinomycetes (Bulgaria, El Salvador, France, Ireland, Italy, Mexico, Romania, United Kingdom), Malaria (Turkey, Swiss, Spain, Portugal, Philippine, Nicaragua, Mexico, Ireland Finland, Belgium, Angola), ameba (Algeria, Angola, France, Hungary, Nicaragua, Poland, Portugal, Romania, Saudi Arabia, Spain, Turkey)
Skin disorders	Onychodystrophy by humidity (Romania, Colombia, Mexico), dermatitis by sunlight (Costa Rica), scleroderma by silica or solvents (Canada, Bulgaria)
Cardio-cerebro-vascular disorders	Ischemic heart disease by increased strain and other physical and neuropsychological burdens (Romania, Korea), myocardial infarction, dissection aneurysm, subarachnoid hemorrhage, and cerebral hemorrhage by psychological stress (Korea, Japan), sudden death by severe psychological stress (Korea, Japan), hypertension by neuropsychological stress (Romania), cardiovascular disorder by psychological stress (Philippines)
Mental, behavioral disorders	Neurosis by long-term direct service to people (Russia, Romania, Mexico)
Disorders of the digestive system	Peptic ulcer and intestinal hernia by psychological stress (Philippines)
Congenital disorders	Congenital viral infection, hydrocephalus, microcephalia, retarded development, skin change, premature birth, inflammation, low weight at birth (Denmark)
Neurological disorders	Toxic autonomic neuropathy by esters, vinyl chloride, unsaturated aliphatic hydrocarbons, carbon monoxide, and vibration (Bulgaria)
Eye disorders	Ophthalmia by electrical light (China), chemical burn of eyes (China), occupational cataracts (31 countries)*, snow blindness (India)
Musculoskeletal disorders	Chronic low back pain (Bulgaria, Denmark,) chronic disorders of the lumbar spine (France, German, European Union, Spain, Belgium), herniated lumbar disc (Italy), intervertebral disc displacement (Taiwan), vertebral degeneration and back pain and neck and disc changes (Saudi Arabia)

2,3,7,8-TCDD: 2,3,7,8-tetrachlorodibenzodioxin, *: Algeria, Angola, Australia, Austria, Bangladesh, Bulgaria, Belgium, Canada, Costa Rica, Colombia, Denmark, El Salvador, France, Hong Kong, Hungary, India, Ireland, Italy, Japan, Korea, Malaysia, Mexico, Philippine, Poland, Portugal, Romania, Singapore, Slovakia, Turkey, United Kingdom.

## Conclusion

Since the original establishment of the ILO List of Occupational Diseases in 1925 (C18) with 3 occupational diseases [13], the list has played a key role in harmonizing the development of policies on occupational diseases and in promoting their prevention at the international level. The ILO List of Occupational Diseases has been updated continually, reflecting changes in the structure of modern industry and the scientific advancement of occupational medicine. In addition, as an agreement of the tripartite meetings and approved results of the International Labour Conference, the ILO list of occupational disease should have been in the common territory of workers, employers, and governments. Until C121 was revised in 1980, the ILO List of Occupational Diseases had been an appended schedule for a workers’ compensation scheme, in which the included items were limited. Since R194, the ILO List has changed to a focus on prevention, recording, and reporting of occupational diseases by expanding its scope to occupational cancer, several musculoskeletal diseases, and mental and behavioral diseases. Despite the increased number of items in R194, cardiovascular disorders related to overwork and low back pain, among the primary compensable diseases in some countries such as, Denmark [44], Japan [45], and Korea [46], are not included, further implying that the ILO List cannot effectively represent the national workers’ compensation system of each of its member states. Driscoll et al. [5] suggested three criteria for a compensable list of occupational diseases: strong scientific evidence, clear diagnostic criteria, and a large proportion of cases, and on this basis they ruled out low back pain and ischemic heart disorders from the recommended compensable list. Despite the importance of low back pain and cardiovascular disorders in occupational health from a preventive perspective, the inclusion of them into a compensable occupational disease list may not be the most effective policy for the health management of these disorders.

According to paragraphs 3–5 of R194, the list should regularly be reviewed by the ILO, and each member state should communicate its national list of occupational diseases to the ILO. Despite the increase in content based on ongoing discussion at tripartite meetings, the ILO List in R194 has some limitations, such as a general description of occupational carcinogens without specific organ sites, relatively limited items in the lists of risk factors and disorders, a lack of definition of the disease or exposure, and a slow revision process.

Future revision of the ILO List of Occupational Diseases will require regular discussion considering including not only the latest knowledge on occupational diseases from evidence-based medicine, such as recent reports from the IARC [47], but also comprehensive information regarding updated national lists from member states.

Among the 50 member states that had provided their national occupational disease lists to prepare the new ILO list in 2005, until 2012, 30 of these countries had lists with a structure similar to that of the ILO List in R194. The ILO List of Occupational Diseases is important for the development of national strategies for occupational disease prevention. However, it cannot reflect all aspects of the compensation systems of the member countries, which have been developed to accompany their own social security systems. Each stage in the development of the List of Occupational Diseases in the ILO has been influenced by the policies on occupational health of the day, from the Industrial Revolution to the present. Development or revision of the national occupational disease lists of each country may need to take the lead in developing the workers’ compensation part of a more appropriate social security system.

## Summary

Tracing the historical changes of the ILO List of Occupational Diseases reflects the context of industrial and social change. The three occupational diseases (anthrax, lead poisoning, and mercury poisoning) in the first ILO List of Occupational Diseases, set up in 1925 as a workmen’s compensation convention, reflected the growth in the textile industry, particularly woolen mills and the growing demand for lead and mercury from the Industrial Revolution. Until the 1960s, 10 occupational diseases (9 types of industrial poisoning and 1 infection) were representative compensable occupational diseases listed in C121 (1964), which implies that occupational diseases in this era were equated with industrial poisoning. Since 1980, due to scientific advancements in diagnostic techniques and medical science, noise-induced hearing loss and several bronchopulmonary diseases have been incorporated into the ILO List with an additional list of chemicals (C121, 1980). Although this change reflected the era of broadening the focus on occupational disease beyond occupational poisoning and the number of occupational disease list was increased to 28 items from 10 items, the ILO List was still in the scope of workers’ compensation conventions up to that point. Since 2002, changes in the structure of industry, emerging new chemicals, and advancements in national workers compensation schemes in several developed countries have provoked the ILO to revise its occupational disease list. An expert group reviewed scientific evidence and conducted a questionnaire survey of the member states. The recommendation was a comprehensive reconstruction of the occupational disease list in a new format (causes: chemical, physical and biological agents; diseases: pulmonary, skin, musculoskeletal, and cancer). The ILO List adopted in 2002 had been revised through two tripartite meetings in 2005 and 2009, taking into account the perspectives of labor, management, and government representatives. The latest version of the ILO List of Occupational Diseases adopted in 2010 will go through an ongoing regular revision process in order to incorporate recent scientific developments. Among the 50 member states that had provided their national lists of occupational diseases to prepare the new ILO List in 2005, until 2012, 30 countries were found to have an occupational disease list with a similar structure to the ILO List in R194. As stated in the preface of R194 [36], the ILO List of Occupational Diseases now provides a comprehensive guideline for the prevention of occupational diseases, rather than a minimal compensable list for the reference of national compensation systems.

## Competing interests

The authors declare that they have no competing interests.

## Authors’ contributions

EA Kim reviewed the historical change of ILO occupational disease list. SK Kang contributed all the review process. Both authors read and approved the final manuscript.
